# Secukinumab leads to shifts from stage-based towards response-based disease clusters—comparative data from very early and established psoriatic arthritis

**DOI:** 10.1186/s13075-020-02268-y

**Published:** 2020-09-09

**Authors:** Eleni Kampylafka, Koray Tascilar, Veronika Lerchen, Christina Linz, Maria Sokolova, Ana Zekovic, Arnd Kleyer, David Simon, Jürgen Rech, Michael Sticherling, Georg Schett, Axel J. Hueber

**Affiliations:** 1 Department of Internal Medicine 3 - Rheumatology and Immunology, Friedrich-Alexander-University Erlangen-Nuremberg and Universitätsklinikum Erlangen, Ulmenweg 18, 91054 Erlangen, Germany; 2 Deutsches Zentrum für Immuntherapie, Friedrich-Alexander-University Erlangen-Nuremberg and Universitätsklinikum Erlangen, Erlangen, Germany; 3 Department of Dermatology, Friedrich-Alexander-University Erlangen-Nuremberg and Universitätsklinikum Erlangen, Erlangen, Germany; 4 Rheumatology Section, Sozialstiftung Bamberg, Bamberg, Germany

**Keywords:** Psoriatic arthritis, Secukinumab, IL-17A, Quality of life, Cluster analysis

## Abstract

**Background:**

Limited information exists about the very early forms of psoriatic arthritis. In particular, differences and responsiveness of patient-reported outcomes (PROs) in very early as compared to established PsA have not been investigated to date.

**Methods:**

Cross-sectional and prospective longitudinal evaluation of PROs related to pain (VAS), physical function (HAQ-DI, SF-36 physical), mental function (SF-36 mental), impact of psoriatic skin (DLQI), joint (PsAID), and global disease (VAS) in two small prospective observational studies on secukinumab 300 mg over 6 months in very early disease patients (IVEPSA study, *N* = 20) and established PsA (PSARTROS study, *N* = 20). Cluster analysis was performed at baseline and 24-weeks of follow-up.

**Results:**

While responses in pain and physical activity-related PROs to secukinumab were more pronounced in established PsA than a very early disease, effects on PROs related to general health perception, as well as those related to emotional and mental well-being, were modified in a similar way in very early disease and established PsA. Cluster analysis based on global disease activity and PROs showed that baseline clusters reflected very early disease and established PsA, while after secukinumab treatment these clusters were abolished and new clusters based on differential responses to physically and mentally oriented PROs formed.

**Conclusions:**

Inhibition of IL-17A by secukinumab leads to comprehensive improvement of general health perception and mental well-being in very early and established PsA, while overall responses in pain and physical activity are more pronounced in established disease. Most importantly, treatment restructures the original patients’ clusters based on disease stage and leads to the formation of new clusters that reflect their response in physical and mental-orientated PROs.

**Trial registration:**

NCT02483234, registered 26 June 2015, retrospectively registered.

## Background

Quality of life (QOL) is considerably impaired in patients with immune-mediated inflammatory disease and improvement of life quality is a central aim of treatment. Psoriatic skin and joint disease both constitute a substantial burden for patients impairing quality of life at multiple levels and being associated with an increased risk of depression [1]. Therefore, aside from measuring objective manifestations of psoriatic skin (e.g., PASI) and/or joint (DAPSA) disease, patient-reported outcomes (PROs) are valuable tools to complement the vision on the effect of disease on QOL and the potential of therapy to improve QOL [2]. PROs used in psoriatic disease are visual analog scales (VAS) for pain and global disease activity and function and quality of life questionnaires. Among the latter, the 36-Item Short Form Survey (SF-36) and the Health Assessment Questionnaire Disability Index (HAQ-DI) were not initially developed for PsA patients and may thus not fully reflect the influence of PsA on the health status of the patients. Additional indices such as the Psoriatic Arthritis Impact of Disease Questionnaire (PsAID) were specifically developed for PsA [3], while the Dermatology Life Quality Index (DLQI) was created with an input from several different skin diseases, including psoriasis [4].

The use of PROs in the evaluation of psoriatic disease can give additional insights, since physician-based outcome measures prioritize the biomedical dimension of diseases while not fully incorporating the patients’ experience. Several interesting research perspectives emerge from this situation such as the possibility to cluster disease endotypes based on the impact of the disease for the patients. Furthermore, PRO may allow a better understanding of the very early psoriatic disease by defining potential patterns that reflect the impact of the early disease on the patients. In previous experiments, we have characterized in detail the impact of IL-17A inhibition by secukinumab in very early and established PsA. In these two small in-depth studies (PSARTROS and IVEPSA [5, 6]), we prospectively collected an array of PROs that allows us to compare the impact of very early and established PsA on the patients and to test the effect of an effective therapeutic intervention strategy in this respect. While the principle efficacy of IL-17A inhibition on PROs has been demonstrated in post hoc analyses of phase 3 studies [7–9], no data are available that looked at very early PsA and compared these data with established disease. Hence, the aim of this study was to compare the effect of IL-17A inhibition by secukinumab on QOL of patients with very early and established PsA and to explore whether such intervention leads to a re-clustering of patients based on the impact of the disease on QOL.

## Methods

### Study population

Patients from the IVEPsA study [6] on very early PsA (*N* = 20) and the PSARTROS [5] study on established PsA (*N* = 20) were included in this analysis. The very early PsA patients of the IVEPsA study were required to fulfill the following criteria: (i) presence of moderate to severe psoriasis (PASI score > 6) or scalp or nail involvement; (ii) absence of present or past joint swelling, active enthesitis, or dactylitis; and (iii) presence of subclinical inflammatory changes in magnetic resonance imaging (MRI) or erosive changes in MRI or high-resolution peripheral quantitative computed tomography (HR-pQCT). Established PsA patients of the PSARTROS study had to fulfill the CASPAR classification criteria of PsA, had to have symptoms of PsA for at least 6 months prior to inclusion into the study, and had to have an active joint disease with at least 3 tender and 3 swollen joints on the 78/76 joint count.

Corticosteroid treatment was allowed if at stable doses of ≤ 10 mg/day prednisone for at least 2 weeks before baseline and had to remain on a stable dose until the end of the study. Concomitant non-biologic treatment was allowed if on a stable dose for at least 4 weeks before baseline and throughout the study. Previous TNF inhibitor therapy was allowed after appropriate washout periods. Patients, who had previously used drugs targeting IL-17 or IL-23p40, were not eligible to participate in either of the studies. Other conditions such as pregnancy, recent live vaccines, history of tuberculosis, use of opioid analgesics, history of alcohol, or drug abuse within the last 6 months or any uncontrolled medical condition were not allowed. All patients provided written informed consent, and the institutional review board of the University Clinic Erlangen approved the study.

### Study procedures and data collection

All patients received secukinumab treatment 300 mg sc. once weekly for the first 4 weeks and once monthly thereafter for a total period of 24 weeks. All patients were longitudinally assessed for 78 tender joint counts (TJC) and 76 swollen joint counts (SJC), the severity of pain and the perceived global disease activity using visual analog scales (VAS), composite disease activity measures for joint (Disease Activity Score 28, DAS-28; Disease Activity Score in Psoriatic Arthritis, DAPSA) and skin disease (total Psoriasis Area and Severity Index, PASI; Body Surface Area, BSA), and several common and disease-specific PROs (SF-36, DLQI, PsAID, and HAQ-DI) at baseline and after 4, 12, and 24 weeks.

### Statistical analysis

Baseline study characteristics were tabulated using appropriate summary statistics. The course of patient-reported outcomes and quality of life indicators were plotted as mean scores and 95% confidence intervals over study visits. Changes from baseline values were estimated using linear mixed effects models adjusted for baseline values of each scale, gender, age and disease duration, and plotted as model coefficients and respective 95% confidence intervals that represent adjusted mean absolute improvement from baseline. Scale signs were inverted as necessary to ease interpretability, age, disease duration, and baseline values were centered at their mean. Study visit week was included in the model as a categorical variable using the baseline visit as reference and differences of changes over time by study cohort were assessed using time-cohort interaction terms.

We conducted a cluster analysis of study participants by patient-reported outcomes and overall disease assessment. The scores were centered around their mean and scaled by their standard deviations to generate heat maps, and Euclidean distances were used for k-means clustering to generate marginal dendrograms on the patient and measurement axes. Further subgroup analyses were conducted using the Mann-Whitney *U* test for comparisons of quantitative variables, chi-square test for comparisons of qualitative variables and Spearman’s rank correlation coefficient for relations. Data manipulation and analyses were conducted using R v. 3.5.3 (R Foundation for Statistical Computing, Vienna. Austria). Two-sided *p* values less than 0.05 were considered significant.

## Results

Out of the 40 initially included patients, 19/20 very early PsA patients and 17/20 established PsA patients completed the study. One patient dropped out due to consent withdrawal, two due to lack of efficacy and one due to recurrent pharyngitis. The descriptive and baseline characteristics of the patients are presented in Table 1.

**Table 1:** Descriptives and baseline characteristics

	All patients (*n* = 36)	Established PsA (*n* = 17)	Very Early Disease (*n* = 19)	SMD (Established PsA vs. Very Early Disease )
**Demographics**				
Age (*m* ± SD)	50.2 ± 11.5	52.1 ± 9	48.6 ± 13.4	0.304
Sex (male, %)	58.3%	41.2%	73.7%	0.696
Disease duration (*m* ± SD)	10.3 ± 9.4	6.5 ± 5.1	13.9 ± 11.2	0.854
**Therapy**				
Previous DMARDs (%)	52.8%	82.4%	26.3%	0.696
cDMARDs (%)	22.2%	47.1%	0%	1.333
Previous biologics (%)	25%	47.1%	0%	1.081
**Clinical characteristics**				
VAS pain (*m* ± SD)	45.4 ± 27.4	64.1 ± 17.5	28.8 ± 23.8	1.686
VAS global (*m* ± SD)	59.4 ± 22.2	57.4 ± 23.6	61.3 ± 21.3	0.177
VAS physician (*m* ± SD)	37.4 ± 22.3	39.6 ± 15.2	35.4 ± 27.4	0.190
TJC (*m* ± SD)	7.4 ± 8.6	12.5 ± 8.7	2.8 ± 5.2	1.354
SJC (*m* ± SD)	2.4 ± 3.1	5.1 ± 2.5	0 ± 0	2.848
DAS-28 ESR (*m* ± SD)	NA	4.9 ± 1	NA	NA
DAPSA (*m* ± SD)	NA	30.8 ± 11.6	NA	NA
PASI (*m* ± SD)	6.6 ± 8.6	2 ± 4.4	10.7 ± 9.4	1.185
BSA% (*m* ± SD)	9.7 ± 14.9	3.6 ± 11.7	15.2 ± 15.6	0.846
**QoL**				
PsAID (*m* ± SD)	4.4 ± 2.2	5.2 ± 2.1	3.7 ± 2.0	0.744
DLQI (*m* ± SD)	7.8 ± 6.5	5.4 ± 6.7	10.1 ± 5.7	0.770
HAQ-DI (*m* ± SD)	0.5 ± 0.5	0.8 ± 0.5	0.3 ± 0.4	1.141
PF-SF36 (*m* ± SD)	69 ± 27	55.3 ± 23.6	81.3 ± 24.1	1.090
Role physical-SF36 (*m* ± SD)	53.5 ± 38.8	41.2 ± 37.4	64.5 ± 37.6	0.621
Role emotional-SF36 (*m* ± SD)	60.2 ± 44.9	58.8 ± 44.9	61.4 ± 46.2	0.057
SF – SF36 (*m* ± SD)	67.7 ± 25.4	64 ± 25.7	71.1 ± 25.4	0.277
Mental health-SF36 (m ± SD)	60.1 ± 18.5	56 ± 17.1	63.8 ± 19.3	0.427
Vitality-SF36 (*m* ± SD)	43.5 ± 22.3	38.2 ± 22.5	48.2 ± 21.6	0.450
BP-SF36 (*m* ± SD)	46.9 ± 25.9	32.3 ± 20.2	60 ± 23.6	1.261
GH-SF36 (*m* ± SD)	42.2 ± 20.9	35.9 ± 15.4	47.8 ± 23.8	0.593
PCS-SF36 (*m* ± SD)	39.5 ± 11.4	33.5 ± 10.3	44.9 ± 9.7	1.143
MCS-SF36 (*m* ± SD)	43.3 ± 11.8	43.5 ± 11.1	43.2 ± 12.7	0.024

*BP* bodily pain, *BSA* body surface area, *cDMARDS* concomitant disease modifying anti-rheumatic drugs, *DAPSA* disease activity in psoriatic arthritis, *DAS* disease activity score, *DLQI* dermatology life quality index, *DMARDs* disease modifying anti-rheumatic drugs, *GH* general health, *HAQ* health assessment questionnaire, *MCS* mental component summary, *PASI* psoriasis area and severity index, *PCS* physical component summary, *PF* physical functioning, *PsA* psoriatic arthritis, *PsAID* psoriatic arthritis impact of disease, *SD* standard deviation, *SF* social functioning, *SF36* short form 36, *SJC* swollen joint count, *SMD* standard mean difference, *TJC* tender joint count, *VAS* visual analog scale

### Comparative analysis of the effect of secukinumab treatment on PROs in very early and established PsA

**Fig. 1:**
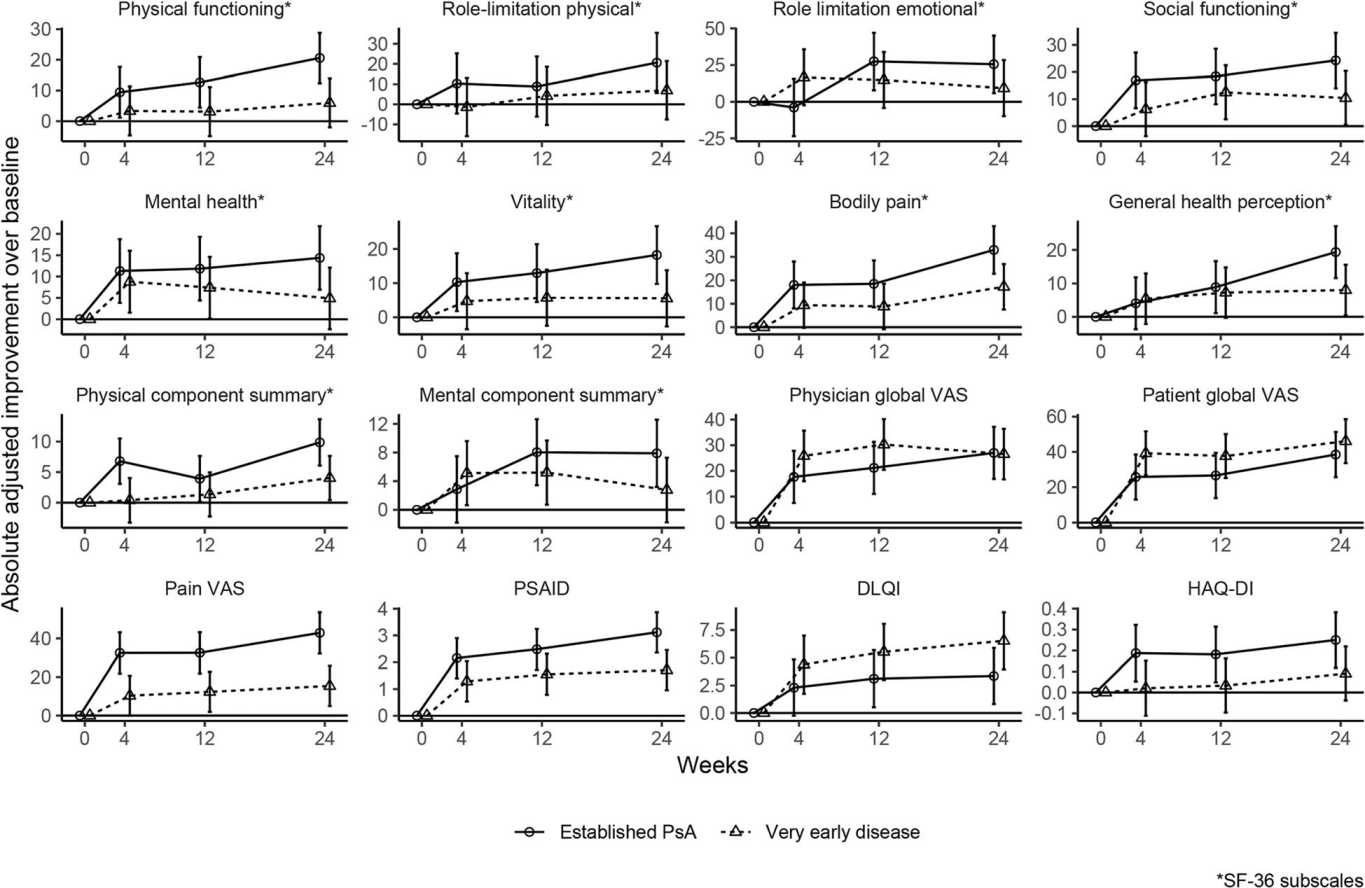
Effects of secukinumab on patient-related outcomes in patients with very early and established psoriatic arthritis. Changes from baseline values adjusted for baseline values of each scale, gender, age and disease duration, and plotted as model coefficients and respective 95% confidence intervals that represent adjusted mean absolute improvement from baseline. DLQI, Dermatology Life Quality Index; HAQ, Health Assessment Questionnaire; PsA, psoriatic arthritis; PsAID, Psoriatic Arthritis Impact Of Disease; VAS, visual analog scale

Rapid improvements within the first 4 weeks of secukinumab treatment were observed in most of the PROs related to QOL of patients with psoriatic disease (Fig. 1). The changes in PROs related to pain and physical function such as pain VAS, HAQ-DI, physical functioning, physical component summary, or physical role limitation were more pronounced in established than in very early PsA, since the burden of joint disease is higher in these individuals. In contrast, more over-reaching PROs, such as the global patient VAS and general health perception, as well as those related to emotional and mental well-being, such as emotional role limitation, social functioning, mental health, and mental component summary were affected in a similar way in very early and established disease. Means and 95% confidence intervals of the respective variables in very early and established disease studies are presented in the supplementary material (Supp. Figure).

### Cluster analysis

**Fig. 2:**
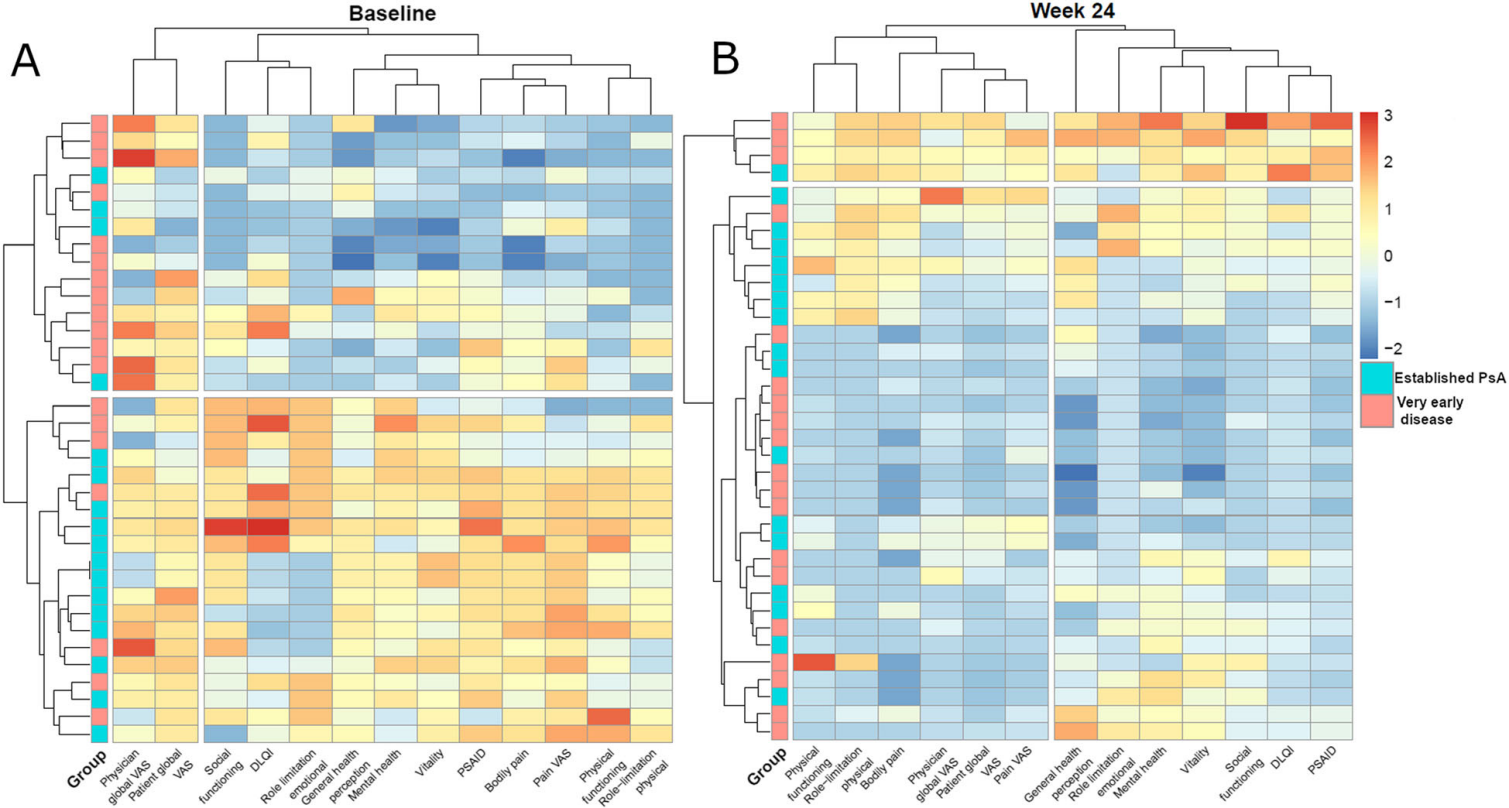
Pre- and post-treatment patient-related outcome-based cluster analysis. Cluster analysis of study participants by patient reported outcomes (PRO) at baseline and at week 24 (exposed to secukinumab 300 mg). Colors show *Z* scores with blue indicating the respective patient-related outcome being better than the mean and red indicating the parameter being lower than mean. Baseline clustering by disease type (very early disease vs. established psoriatic arthritis) is lost after secukinumab treatment and replaced by clusters related to the amount of physical and mental disease burden. Left: Baseline cluster showing accumulation of very early disease patients in the upper cluster with milder PROs, while patients with established disease accumulate in the lower cluster with more severe impact of disease. Right: 24 week clusters showing redistribution of very early and established disease among 4 clusters: top: high disease burden in physical and mental PROs, upper: moderate disease burden in physical and mental PROs, lower: low disease burden in physical and mental PROs, bottom: low disease burden in physical and moderate in mental PROs; DLQI, Dermatology Life Quality Index; PsA, psoriatic arthritis; PsAID, Psoriatic Arthritis Impact of Disease; VAS, visual analog scale

We next performed a cluster analysis to find disease patterns related to PROs in this group of patients and also tested how secukinumab treatment changes these clusters. Results of the cluster analyses at baseline and week 24 are presented as heat-maps and marginal dendrograms in Fig. 2. At baseline, the PRO-based clusters grossly distinguish very early from established PsA. At week 24, however, the baseline distinction between very early from established PsA patients with respect to PROs is no longer visible as the highest level cluster and a small subgroup of 4 patients with overall higher end-of-study scores segregate separately. The two other clusters (on the lower part on the measurement axis) show a separation of measurements that assess the physical dimension of the disease, namely pain, global disease activity, physical functioning, and physical role-limitation as opposed to measurements of the mental dimension, such as social functioning, vitality, mental health, emotional role limitation, or more broader multi-dimensional measurements such as DLQI, PsAID, and general health perception.

### Sub-analyses related to minimal disease activity status and residual imaging signs of inflammation

We also assessed, which of the PROs differ in established PsA patients reaching minimal disease activity (MDA) and in those not reaching this status. Established PsA patients who achieved MDA showed a significantly higher improvement in VAS pain, VAS global disease activity, HAQ-DI, SF-36 physical component score, SF-36 physical functioning, bodily pain, and PsAID (all *p* < 0.05). Residual inflammation (PSAMRIS synovitis and total PSAMRIS at week 24) in very early and established PsA patients correlated with the HAQ-DI at week 24 (*r*_*s*_ = 0.473, *p* = 0.004 and *r*_*s*_ = 0.469, *p* = 0.004). Individual point estimates for established PsA and pre-PsA subgroups were similar but less precise.

## Discussion

These data show that inhibition of IL-17A by secukinumab leads to profound changes in general, physical, and mental health perception of PsA patients in addition to the objective improvement in signs and symptoms and imaging findings of inflammation, which have been reported previously [5, 6]. While the effects of secukinumab on pain and physical function observed in established PsA are not surprising and have already been observed in the large phase 3 studies [7–9], in which ACR responses comprising pain and function are used as outcomes, the substantial effects of secukinumab treatment on other PROs that are not directly related to pain and physical function, such as social functioning, vitality, mental health, and emotional role limitation are interesting and suggest a much deeper modification of the health state of PsA patients than previously considered. Most importantly, this pattern of health state and QOL modification achieved by the intervention in established disease can also be seen in very early disease patients. To date, no data exist in this very early disease population and while their physical burden related to the joint disease is lower than in established PsA, their overall impairment in QOL is similar to established disease, necessitating strategies that allow earlier intervention [10, 11]. These data support the concept that very early treatment of PsA leads to significant improvement in the patients’ overall health state. While these effects are in part related to skin-related QOL (as measured by the DLQI) resembling previously reported effects of secukinumab in psoriasis patients [12, 13], they may likely reflect a more comprehensive control of the impact of all aspects of the disease in the patient. The observed improvements in bodily pain, which may reflect symptoms related to subclinical joint, entheseal, and tendon inflammation illustrates this concept.

Cluster analysis supported the concept that secukinumab treatment leads to a fundamental change in the impact of the disease on patients. Hence, while patients at baseline cluster according to whether they have established PsA or very early disease with no swelling, these clusters are lost after treatment, leading to segregation of patients according to measurements that assess the physical dimension of the disease, as opposed to those for the mental and quality of life dimensions. This change in patient clustering shows that differences in discriminating very early PsA from established PsA with respect to the quality of life, pain, and disease perception at baseline are abolished by treatment and render these groups indistinguishable. Cluster analysis further highlighted the distinct response characteristics of physical and mental dimensions in patients treated with secukinumab with clusters, in which the response in the two domains are aligned or where they diverge, showing good physical response but poorer mental response to treatment. This observation points towards an unmet need in the treatment of psoriatic disease, showing that the physical and mental components of the disease may require individual management strategies. Of note, these findings also impact on how to judge remission in PsA. Even comprehensive instruments like MDA are based on physical components in the judgment of disease activity. Thus, reaching MDA upon secukinumab treatment was associated with physical function-oriented instruments such as HAQ-DI and SF-36 physical function and bodily pain. These data align with the previous observations from different PsA therapies showing that MDA is associated with better QOL outcomes [14, 15].

When interpreting these data, certain limitations need to be considered. First, patient numbers are rather small, which is limiting the power of the study. Hence, the concept of treatment-based cluster re-organization needs to be validated in larger populations. In order to improve the precision of our estimates for between-group comparisons, we were using all baseline and post-baseline measurements of the outcomes in the analysis instead of focusing on measurements at one specific time point. Additionally, the follow-up of 6 months is short, thus not allowing to judge whether these clusters are stable in the mid- and long-term follow-up.

## Conclusions

Altogether, these data show that effective interventions in psoriatic disease lead to a re-clustering of patients’ subgroups based on their PROs and health status. While patients with established PsA and psoriasis patients with a very early form of musculoskeletal disease fundamentally differ in their PROs and cluster differently, effective therapy, like in this case, IL-17A inhibition by secukinumab abolished these clusters and redistributed patients based on their concordant or discordant physical and mental outcomes.

## Supplementary information

**Additional file 1 : Supplementary Figure.** Effects of secukinumab on patient-related outcomes in patients with very early and established psoriatic arthritis. Means and 95% confidence intervals of the respective variables at baseline, 4, 12 and 24 weeks of secukinumab treatment). DLQI, Dermatology Life Quality Index; HAQ, Health Assessment Questionnaire; PsA, psoriatic arthritis; PsAID, Psoriatic Arthritis Impact of Disease; VAS, visual analog scale

## Data Availability

The datasets used and/or analyzed during the current study are available from the corresponding author on reasonable request.

## Abbreviations

BP: Bodily pain
BSA: Body surface area
DAPSA: Disease activity in psoriatic arthritis
DLQI: Dermatology Life Quality Index
IL: Interleukin
GH: General health
HAQ-DI: Health Assessment Questionnaire Disability Index
MCS: Mental component summary
PASI: Psoriasis area severity index
PCS: Physical component summary
PF: Physical functioning
PsAID: Psoriatic Arthritis Impact of Disease Questionnaire
PSA: Psoriatic arthritis
QOL: Quality of life
SF: Social functioning
SF-36: Short Form Survey 36
SJC: Swollen joint count
TJC: Tender joint count
VAS: Visual analog scale
