# Supplementary figures and images for: Secukinumab leads to shifts from stage-based towards response-based disease clusters—comparative data from very early and established psoriatic arthritis
Source: Arthritis Res Ther. 2020 Sep 9;22:207. doi: 10.1186/s13075-020-02268-y (PMC7488266; doi:10.1186/s13075-020-02268-y)

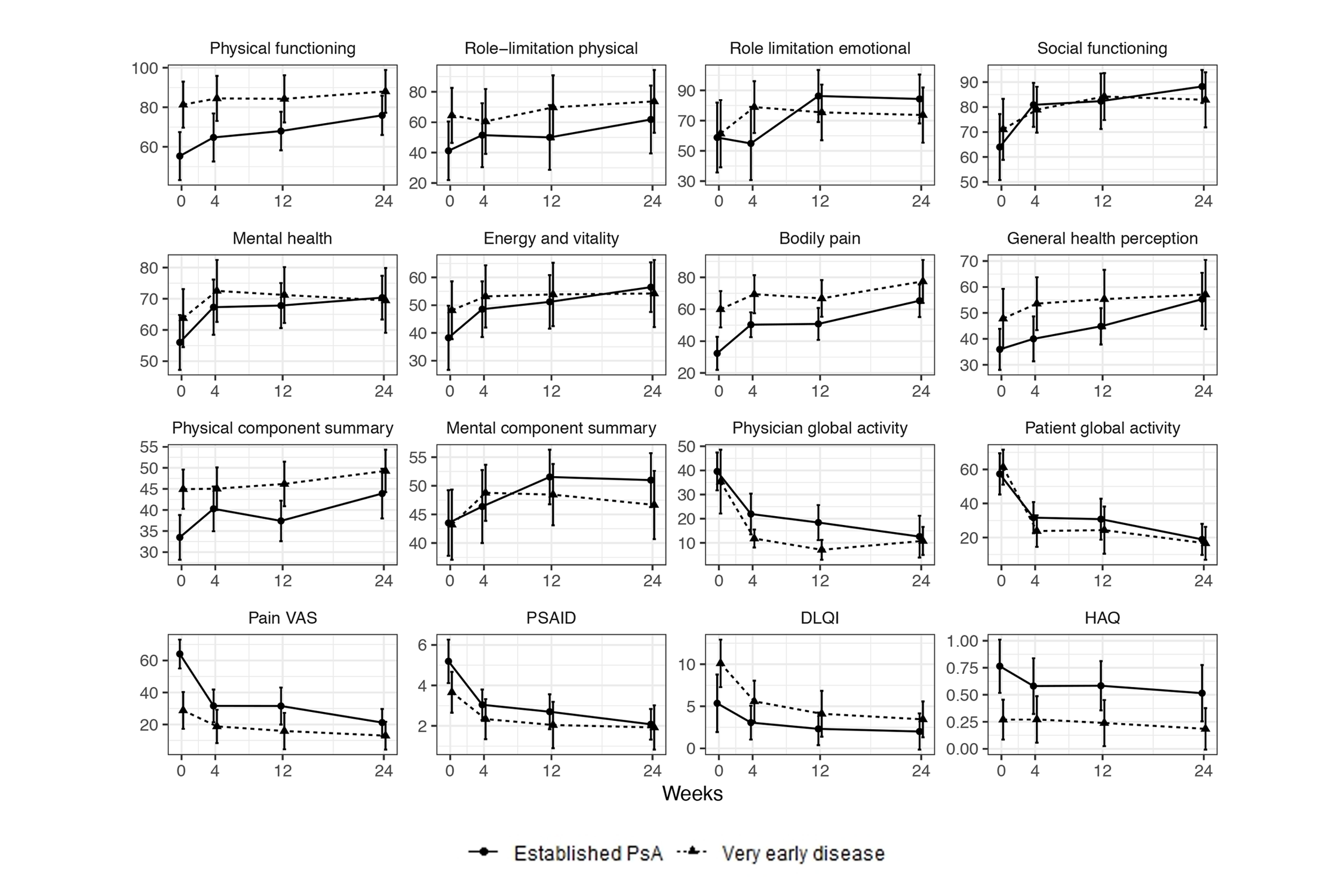

Supplement: Supplementary file 1 — Additional file 1 : Supplementary Figure. Effects of secukinumab on patient-related outcomes in patients with very early and established psoriatic arthritis. Means and 95% confidence intervals of the respective variables at baseline, 4, 12 and 24 weeks of secukinumab treatment). DLQI, Dermatology Life Quality Index; HAQ, Health Assessment Questionnaire; PsA, psoriatic arthritis; PsAID, Psoriatic Arthritis Impact of Disease; VAS, visual analog scale [file 13075_2020_2268_MOESM1_ESM.tif]
